# Long-term prognosis of curative endoscopic submucosal dissection for early colorectal cancer according to submucosal invasion: a multicenter cohort study

**DOI:** 10.1186/s12876-022-02499-0

**Published:** 2022-09-13

**Authors:** Jongbeom Shin, Eun Ran Kim, Hyun Joo Jang, Dong Hoon Baek, Dong-Hoon Yang, Bo-In Lee, Kwang Bum Cho, Jin Woong Cho, Sung-Ae Jung, Su Jin Hong, Bong Min Ko, The Research Group for Endoscopic Submucosal Dissection in Korean Society of Gastrointestinal Endoscopy, Jung-Won Jeon, Weon Jin Ko, Sun Moon Kim, Young Dae Kim, Kim Chan Gyoo, Gwang Ho Baik, In Kyung Yoo, Lee Kang Nyeong, Si Hyung Lee, Chul-Hyun Lim, Seong Woo Jeon

**Affiliations:** 1 Division of Gastroenterology, Department of Internal Medicine, Inha University School of Medicine, Incheon, Korea; 2 Department of Internal Medicine, Samsung Medical Center, Sungkyunkwan University School of Medicine, 81 Irwon-ro, Gangnam-gu, Seoul, 06351 Korea; 3 Division of Gastroenterology and Hepatology, Department of Medicine, Dongtan Sacred Heart Hospital, Hallym University School of Medicine, Hwaseong, Korea; 4 Division of Gastoenterology, Department of Internal Medicine, Pusan National University School of Medicine, Pusan National University Hospital, Yangsan, Korea; 5 Department of Gastroenterology, Asan Medical Center, University of Ulsan College of Medicine, Seoul, Korea; 6 Division of Gastroenterology, Department of Internal Medicine, Seoul St. Mary’s Hospital, College of Medicine, The Catholic University of Korea, Seoul, Korea; 7 Division of Gastroenterology and Hepatology, Department of Internal Medicine, Dongsan Medical Center, Keimyung University School of Medicine, Daegu, Korea; 8 Department of Internal Medicine, Presbyterian Medical Center, Jeonju, Korea; 9 Department of Internal Medicine, College of Medicine, Ewha Womans University, Seoul, Korea; 10 Department of Internal Medicine, Digestive Disease Center and Research Institute, Soonchunhyang University School of Medicine, Soonchunhyang University Bucheon Hospital, 170 Jomaru-ro, Bucheon, 14584 Gyeongkido Korea; 11 Korean Society of Gastrointestinal Endoscopy, 156 Yanghwa-ro, Mapo-gu, Seoul, 04050 Korea

**Keywords:** Early colorectal cancer, Endoscopic submucosal dissection, Recurrence free survival

## Abstract

**Background:**

Endoscopic submucosal dissection (ESD) can provide a high en bloc resection rate and has been widely applied as curative treatment for early colorectal cancer (ECC). However, surgical treatment is occasionally required, and reports on the long-term prognosis of ESD are insufficient. This study aimed to investigate the long-term outcomes of ECC removal by ESD, including local recurrence and metastasis.

**Methods:**

This multicenter study was conducted retrospectively on 450 consecutive patients with ECC who were treated with ESD between November 2003 and December 2013. Clinical, pathological, and endoscopic data were collected to determine tumor depth, resection margin, lymphovascular invasion, and recurrence.

**Results:**

The median follow-up period was 53.8 (12–138 months). The en bloc resection rate was 85.3% (384) and in intramucosal cancer being 84.1% and in superficial submucosal invasion (SM1) cancer being 89.8% (*p* = 0.158). The curative resection rate was 76.0% (n = 342), and there was no statistical difference between the two groups (77.3% vs. 71.4%, *p* = 0.231). The overall recurrence free survival rate (RFS) was 98.7% (444/450). In patients with curative resection, there was no statistically significant difference in RFS according to invasion depth (intramucosal: 99.3% vs. SM1: 97.1%, *p* = 0.248).

**Conclusions:**

Patients with curatively resected ECC treated with ESD showed favorable long-term outcomes. Curatively resected SM1 cancer has a RFS similar to that of intramucosal cancer.

## Background

Colorectal cancer (CRC) is the third most common cancer and the fourth leading cause of cancer mortality worldwide [1]. The CRC is predicted to cause 2.2 million new cases and 1.1 million deaths by 2030 [2]. Since most CRCs originate from colon adenoma [3], screening by performing colonoscopy to identify early and resect these adenomas plays an important role in cancer prevention [4]. Recently, with the implementation of CRC screening programs, there is an increase and the widespread use of high-resolution endoscopes [5]. Hence, it is predicted that patients would be diagnosed with early stage of CRC more frequently [6].

The prognosis of patients with CRC is rudimentary, as determined by the stage of the cancer at the time of diagnosis. According to the recent data, nearly 75% of newly diagnosed CRC is in early stage of cancer without metastasis [7, 8]. Early CRC (ECC) is defined as cancer cells that are confined to the mucosa and submucosa with or without metastasis to lymph nodes or other organs [9–11] An ECC can be completely removed surgically, the 5-year survival rate is greater than 90%, and the prognosis is satisfactory [12, 13].

The risk of complications in patients undergoing traditional surgical resection is high (20%) [14]; hence, the demand for endoscopic resection is increasing. Recently, endoscopic resection, which can be selectively employed for stage I cancer, has been shown to significantly reduce the risk of complications [15, 16]. Endoscopic submucosal dissection (ESD) can provide a high en bloc resection rate and is widely accepted as a treatment for ECC [17–20].

According to the Japanese Society for Cancer of the Colon and Rectum (JSCCR) 2019 guidelines, intramucosal or superficial submucosal invasive (SM1) CRC (defined by the depth of invasion from the muscularis mucosa as less than 1,000 um) is an indication for endoscopic resection [21].

There is an increase in the number of studies on the outcomes of ESD; however, data on the long-term outcomes of ESD for ECC are insufficient. This is because the results of previous studies were confined to pedunculated polyp lesions [22], patients with high-grade dysplasia and adenomas accounted for the majority of the study subjects [23–25], or the number was not sufficiently large [26]. Particularly, the data on the long-term outcomes of SM1 cancer removal by ESD is insufficient.

Hence, this study aimed to identify the long-term outcomes of ESD for superficial ECC, including recurrences and metastasis, and to evaluate the differences in the pathologic features for risk assessment of recurrence according to the submucosal invasion.

## Materials and methods

### Study subjects

**Fig. 1:**
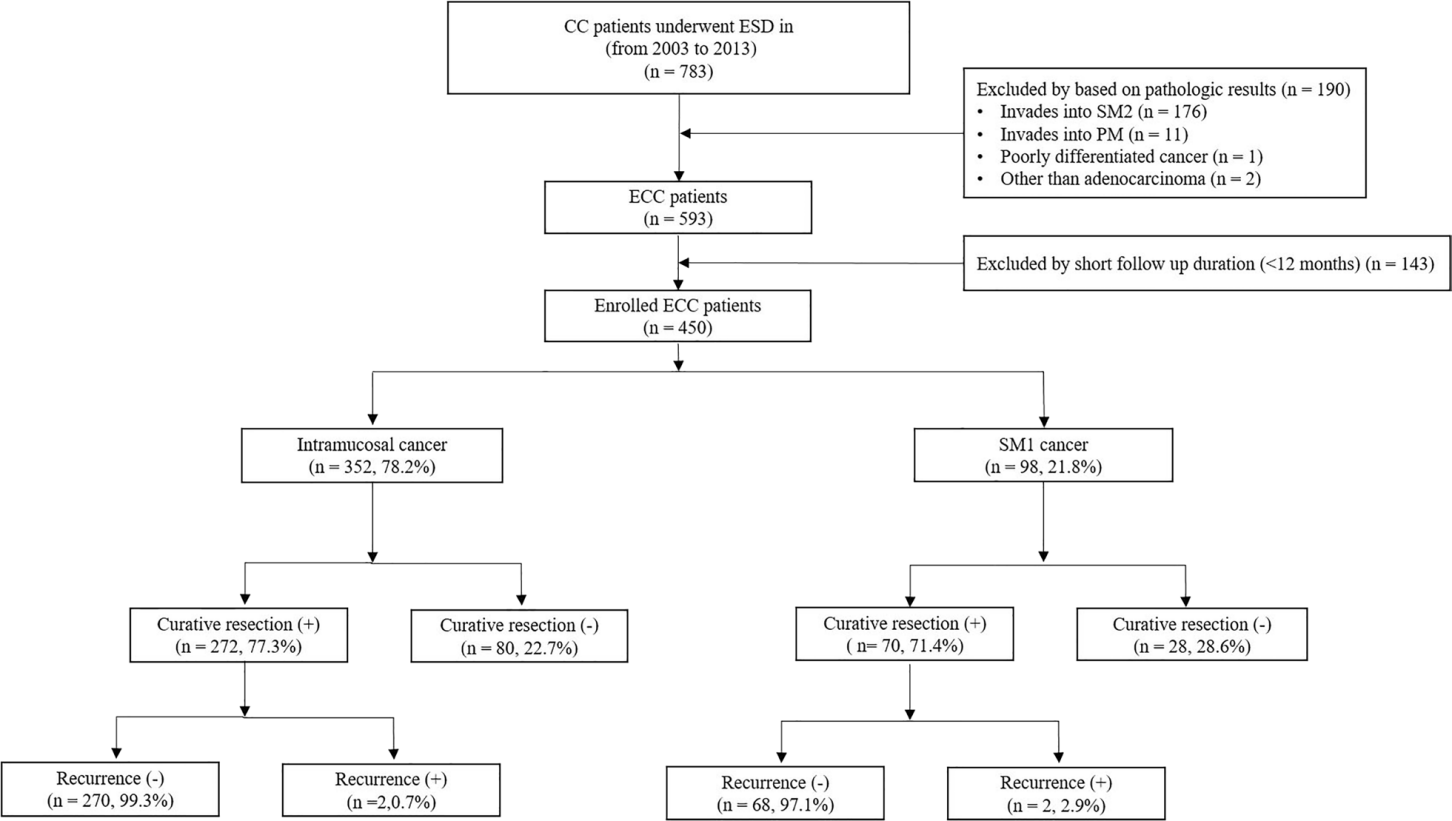
Patient selection flowchart. Abbreviations: *ESD* endoscopic submucosal dissection, *ECC* early colorectal cancer, *SM* submucosa, *PM* proper muscle

Between November 2003 and December 2013, a total of 783 patients who underwent ESD for CRC at nine Korean ESD study group-affiliated hospitals (Asan Medical Center, Hallym University Medical Center, Presbyterian Medical Center, Samsung Medical Center, Kyungpook National University Medical Center, Keimyung University Dongsan Medical Center, Seoul St. Mary’s Hospital, Ewha Woman’s University Medical Center, and Incheon St. Mary’s Hospital) were considered for enrollment in this study. The patients were retrospectively selected according to the following inclusion criteria: (1) age > 20 years, (2) sufficient pathological reports, and (3) no history of previous or synchronous cancer. Based on the following criteria, 333 patients were excluded: (1) deep submucosal invasion (≥ 1000 um, SM2) in the pathological results, (2) in the case of poorly differentiated carcinoma or carcinoma other than adenocarcinoma, and (3) follow-up for less than 12 months and incomplete data. The remaining 450 patients were analyzed in this retrospective study (Fig. 1). The medical records of all patients were reviewed to obtain clinical information. The patients were allocated by the depth of cancer invasion into intramucosal cancer (n = 352, 78.2%) and SM1 cancer (n = 98, 21.8%). For the purpose of analysis, the patients were divided into two groups according to curative resection. This study was conducted in accordance with the ethical guidelines of the Declaration of Helsinki and its later amendments, and was approved by the institutional review boards of all the participating hospitals (IRB No. 2015-06-074-002).

### ESD procedure

An ESD for CRC was performed by a gastrointestinal endoscopist who was highly specialized in therapeutic colonoscopy procedures. Written informed consent was obtained from all the patients enrolled in this study. Before performing ESD, the endoscopist estimated the location and size of the CRC and determined whether ESD was applicable. When endoscopic gross morphological findings suggestive of submucosal invasion, such as Kudo’s pit pattern or non-lifting sign, were observed, then surgery was recommended instead of ESD. All ESD procedures were performed using a standard single-accessory-channel endoscope while the patient was under conscious sedation. A mixture of normal saline, glycerin, and/or sodium hyaluronate with a small amount of indigo carmine was injected into the submucosal space using a 21- or 23-gauge needle. Electrosurgical instruments, such as flex, hook, dual, insulated tip (IT), and IT-nano knives, were selected at the discretion of the endoscopist and used for mucosal incision and submucosal dissection. The submucosal layer was directly dissected using one of the designated knives until complete removal was achieved. After ESD, prophylactic endoscopic hemostasis and clipping were performed on the exposed vessels with oozing, bleeding, or damaged muscle layers.

### Histological evaluation of ESD specimens

Resected ESD specimens were retrieved using forceps, suctioning, or nets, stretched to an approximate length, and fixed using anti-rust pins on a Styrofoam surface. The full thickness of each specimen was pinned to identify the depth of invasion and the horizontal margin. After macroscopic observation, the ESD specimens were cut parallel to the direction of the closest margin. Specimens were step-sectioned at 1 or 2 mm intervals and then examined. The depth of submucosal invasion was defined as the distance from the deeper edge of the muscularis mucosa to the deepest invasive portion. When the muscularis mucosa could not be identified, the depth of submucosal invasion was considered as the distance between the tumor surface and the deepest invasive portion. Immunostaining including D2-40 and VB was selectively performed when additional evaluation was required, such as unclear lymphovascular structures. All CRC specimens were histologically evaluated and classified according to the World Health Organization system [27].

In this study, CRC was classified as intramucosal carcinoma, carcinoma with superficial submucosal invasion (< 1000 um) (SM1), carcinoma with deep submucosal invasion (≥ 1000 um) (SM2), and carcinoma with proper muscle invasion (PM).

### Definitions

En bloc resection was defined as cancer resection in one piece without fragmentation [10, 20, 28, 29]. Complete histological resection was defined as negative vertical and horizontal margins. The definition of curative resection in this study meant histologically complete resection with no risk of lymph node metastasis on histological examination of CRC specimen based on the JSCCR guidelines: (1) well or moderate differentiation, (2) negative resection margins, (3) no lymphovascular invasion, (4) tumor budding grade 1 (low), and (5) submucosal invasion depth < 1000 um (SM1) [21]. Recurrence was defined as cases where cancer was observed at the site where prior ECC was removed, lymph node metastasis was confirmed, and metachronous cancer occurred during the follow-up period.

### Surveillance after ESD

All study subjects underwent intensive follow-up. Follow-up colonoscopy was performed at one year for patients who underwent curative resection, and at six months and one year for patients who underwent non-curative resection for tumors showing a histologically positive horizontal and deep margins. All enrolled patients underwent at least one follow-up colonoscopy. For early detection of recurrence, computed tomography (CT) scan were recommended once every 1–2 years for mucosal cancer and once every 6–12 months for SM cancer. Tumor marker tests (eg, cancer embryonic antigen) were tested annually only in some cases because follow-up is not recommended in early colorectal cancer below stage I. To evaluate the long-term outcomes, retrospective analysis of the recurrence-free survival (RFS), recurrence, local recurrence, and metachronous occurrence rates was performed.

### Statistical analyses

The primary study endpoint was RFS in patients with intramucosal cancer and SM1 cancer removed using ESD. The clinical characteristics of the study subjects are expressed as medians (ranges) for continuous variables and numbers (percentages) for categorical variables. The differences between categorical and continuous variables were analyzed using the Mann–Whitney U test, Student’s *t*-test, *chi-*squared test, or Fisher’s exact test. RFS rates were estimated using the Kaplan–Meier method. Differences in RFS curves among groups were assessed using the log-rank test. Two-tailed *p*-values of < 0.05 were considered statistically significant. Statistical analyses were performed using the SPSS version 25.0 (IBM Corp. (Released 2017), IBM SPSS Statistics for Windows; Armonk, NY, USA).

## Results

### Baseline clinical characteristics

A total of 450 patients (tumor stage 0–I, CRC with < 1000 um submucosal invasion) analyzed, including 271 men (60.2%), and the mean age of the patients was 61.2 ± 10.3 years. Concomitant chronic diseases included hypertension (28.9%), cardiovascular disease (4.2%), diabetes (11.1%), liver cirrhosis (6.4%), and renal insufficiency (1.1%). The patients on antiplatelet drugs or anticoagulants were 11.1%. The proportion of patients with cancer located in the rectum was 48.7%, followed by right colon (27.3%), and left colon (24.0%). The median tumor size was 28 mm (8–102). The en bloc resection rate of all patients was 85.3% (384/450), that of intramucosal cancer was 84.1%, of SM1 cancer was 89.8%. There was no statistical difference between the two groups in terms of the en bloc resection rates (*p* = 0.158). There are no statistically significant differences between intramucosal cancer and SM1 cancer in the baseline clinical characteristics except for morphology. The detailed baseline clinical characteristics of the patients are shown in Table 1.

**Table 1:** Baseline clinical characteristics of study subjects

Variables	Total enrolled patients (n = 450)	Intramucosal cancer (n = 352)	SM1 cancer (n = 98)	*P* value
Age (years)	62 (20–84)	61 (20–84)	63 (30–84)	0.842
Gender (Male), n (%)	271 (60.2)	210 (59.7)	61 (62.2)	0.644
Comorbidity, n (%)				
Hypertension	130 (28.9)	91 (25.9)	39 (39.8)	0.190
Cardiovascular disease	19 (4.2)	15 (4.3)	4 (4.1)	0.949
Diabetes	50 (11.1)	39 (11.1)	11 (11.2)	0.948
Liver cirrhosis	29 ( 6.4)	20 (5.7)	9 (9.2)	0.329
Renal insufficiency	5 (1.1)	3 (0.9)	2 (2.0)	0.622
Use of antiplatelet or anticoagulant				0.328
No, n (%)	400 (88.9)	317 (90.1)	84 (85.7)	
Yes, n (%)	50 (11.1)	35 (9.9)	14 (14.3)	
Cancer location, n (%)				0.138
Rectum	219 (48.7)	180 (51.2)	39 (39.8)	
Left colon	108 (24.0)	80 (22.7)	28 (28.6)	
Right colon	123 (27.3)	92 (26.1)	31 (31.6)	
Tumor size (mm)	28 (8–102)	28 (8–102)	26 (8–53)	0.911
Morphology, n (%)				< 0.001
LST-granular	194 (43.1)	173 (49.1)	21 (21.4)	
LST-nongranular	100 (22.2)	64 (18.2)	36 (36.7)	
Non-LST	156 (34.7)	115 (32.7)	41 (41.8)	
En bloc resection, n (%)				0.158
En bloc	384 (85.3)	296 (84.1)	88 (89.8)	
Piecemeal	66 (14.7)	56 (15.9)	10 (10.2)	
Recurrence, n (%)	6 (1.33)	4 (1.13)	2 (2.0)	0.490
Local recurrence	1 (0.22)	1 (0.28)	–	
Regional lymphnode	2 (0.44)	–	2 (2.0)	
Distant metastasis	2 (0.44)	2 (0.57)	–	
Metachronous cancer	1 (0.22)	1 (0.28)	–	
Follow up duration (month)	53.8 (12–138)	51.6 (2–138)	60 (12–122)	0.332

Data are presented as the median (range) or number (percentage)

### Histological characteristics of ECC patients treated with ESD

According to histological assessments, 352 patients (78.2%) had intramucosal cancer and 98 patients (21.8%) had SM1 cancer. (Fig. 1) The number of patients who met the JSCCR criteria for curative resection was 342 (76.0%) (Table 2). Well differentiated adenocarcinomas were identified in 343 patients (76.2%), and moderately differentiated adenocarcinomas were identified in 107 patients (23.8%). Positive lymphatic invasion was observed in nine patients (2.0%), positive vascular invasion was observed in five patients (1.1%), and intermediate tumor budding was observed in two patients (0.4%). The pathological features associated with recurrence were significantly higher in SM1 cancer than in intramucosal cancer. There was no difference in the curative resection rate between the two groups according to the depth of invasion (272/352, 77.3% vs. 70/98, 71.4%; *p* = 0.231).

**Table 2:** Histological features of early colorectal cancer treated with endoscopic submucosal dissection

Variables	Total enrolled patients (n = 450)	Intramucosal cancer (n = 352)	SM1 cancer (n = 98)	*P* value
Histologic type, n (%)				0.789
Well	343 (76.2)	267 (75.8)	76 (77.6)	
Moderate	107 (23.8)	85 (24.1)	22 (22.4)	
Lymphatic invasion, n (%)				0.004*
Negative	441 (98.0)	349 (99.1)	92 (93.9)	
Positive	9 (2.0)	3 (0.9)	6 (6.1)	
Vascular invasion, n (%)				0.009*
Negative	445 (98.9)	351 (99.7)	94 (95.2)	
Positive	5 (1.1)	1 (0.3)	4 (4.8)	
Tumor budding, n (%)				0.049*
Low	448 (99.6)	352 (100.0)	96 (98.0)	
Intermediate	2 (0.4)	0 (0.0)	2 (2.0)	
Histological depth, n (%)				
Intramucosa	352 (78.2)	352 (100.0)		
SM1 (< 1000 µm)	98 (21.8)		98 (100.0)	
Resection margin, n (%)				
Clear resection margin	351 (78.0)	275 (61.1)	77 (78.6)	0.925
Positive resection margin	99 (22.0)	77 (21.9)	21 (21.4)	
Curative resection, n (%)	342 (76.0)	272 (77.3)	70 (71.4)	0.231

Data are presented as the median (range) or number (percentage)

*Fisher’s exact test

### Long-term outcomes after ESD in CRC patients

**Fig. 2:**
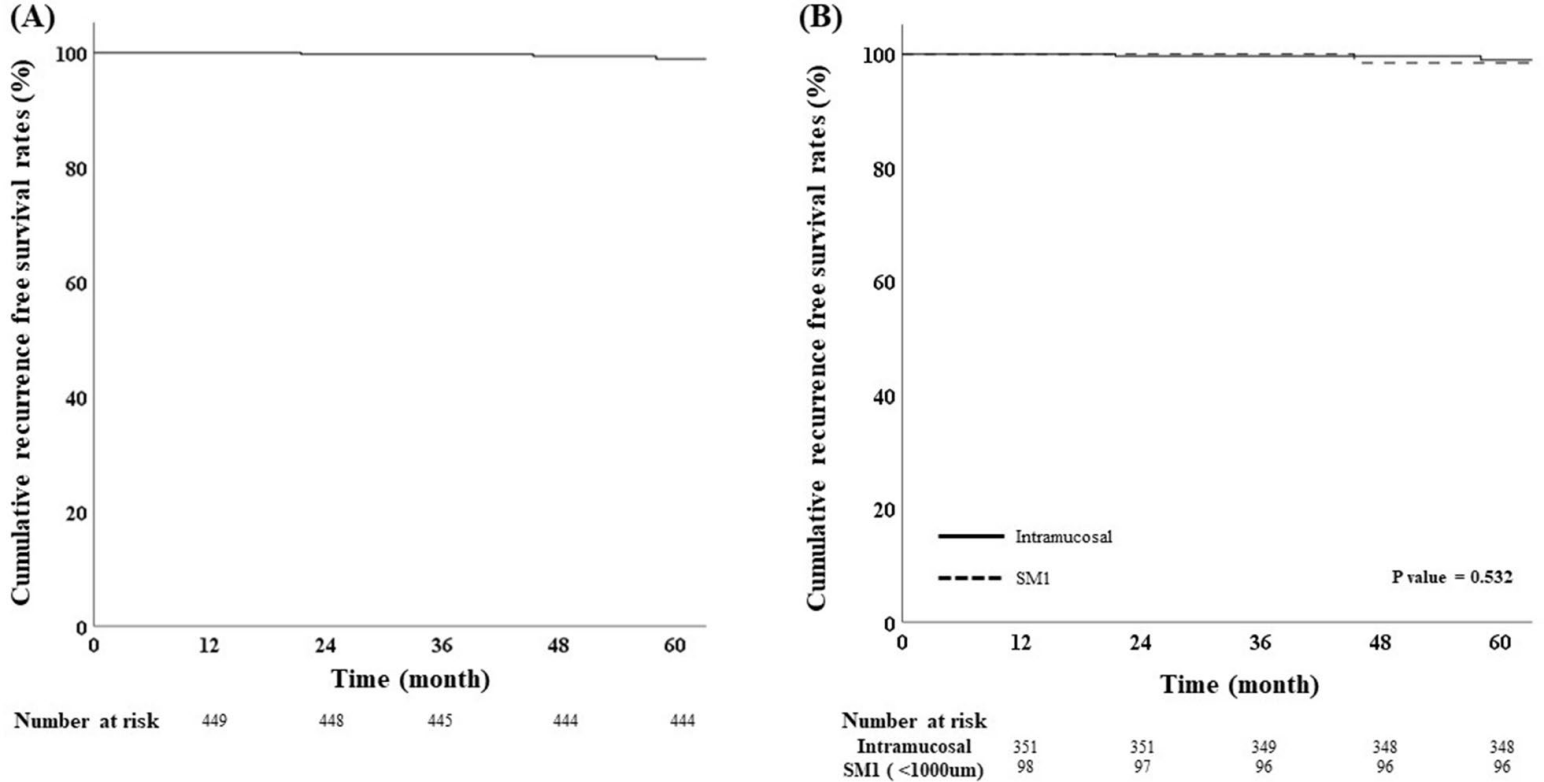
Kaplan–Meier curves demonstrating cumulative recurrence free survival rates in **A** overall enrolled ECC patients and **B** according to depth of cancer invasion

**Fig. 3:**
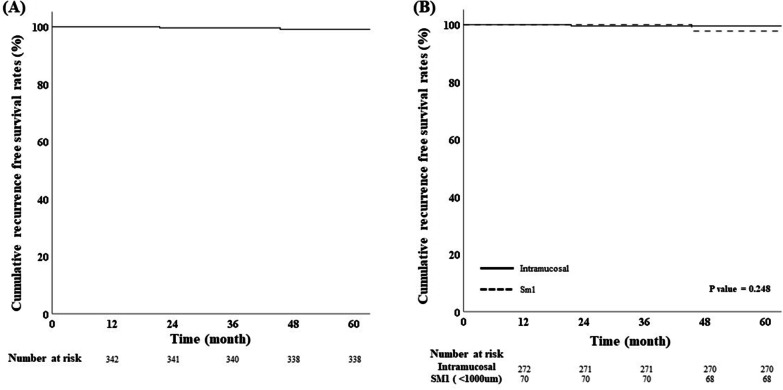
Kaplan–Meier curves demonstrating cumulative recurrence free survival rates **A** in curatively resected ECC patients and **B** according to depth in curatively resected patients

The median follow-up period was 53.8 (12–138 months). The RFS rates were 98.7% (444/450) in all enrolled patients (Fig. 2A) and in curatively resected patients were 98.8%, 4 patients (1.2%) had recurrence. The RFS rate showed no statistically significant differences with respect to depth of invasion (*p* = 0.532) (Fig. 2B). A total of 4 recurrences were observed among the curatively resected patients (Fig. 3A), and two cases of recurrence were observed in each group (Fig. 3B). In intramucosal cancer, one distant metastasis and one metachronous cancer were detected, and in SM1 cancer, two distant lymph node metastases were detected during the follow-up period. In one case of SM1 cancer recurrence, the vertical margin was 1500 μm and the safety margin was 1225 μm, and in another case, the vertical margin was 600 μm and the safety margin was only 300 μm. The clinical and histological features of the recurrence after ESD are shown in Table 3. In 143 patients with a follow-up period of less than 1 year, there was no recurrence except for one case in which cancer was additionally confirmed at a site different from the site removed by ESD at the time of diagnosis. Non-curatively resected patients underwent additional surgical treatment or were closely observed. Among non-curatively resected intramucosal cancer, only 5 (6.25%) underwent additional surgery, and among submucosal cancer, 12 (42.9%) underwent additional surgery. No recurrence was observed in patients who underwent additional surgery, but recurrence was confirmed in 2 patients who underwent close observation.

**Table 3:** Clinical features of the recurrence cases

Case	Sex/age (years)	Location	Size (mm)	Depth of invasion (um)	Vertical resection margin (um) (safety margin, (um))	Resection margin	En bloc resection	ESD related complications	Time to recurrence (month)	Type of recurrence	Treatment for recurrence
Curatively resected patients											
A	M/49	Rectum	24	Intramucosa		Negative	Piecemeal	No	46	Distant metastasis (Lung)	CTx
B	F/70	Rectum	11	SM 300	600 (300)	Negative	En bloc	No	67	Regional LN meta	Surgery + CTx
C	M/70	Rectum	48	Intramucosa		Negative	En bloc	No	21	Meta-chronous cancer	Surgery
D	M/74	Transverse colon	13	SM 275	1500 (1225)	Negative	En bloc	No	45	Regional LN meta	Surgery + CTx
Non curatively resected patients											
E	F/75	Rectum	34	Intramucosa		Positive	En bloc	Perforation	6	Local recurrence	Surgery
F	M/50	Rectum	65	Intramucosa		Positive	En bloc	No	43	Distant metastasis (Liver)	Surgery + CTx

## Discussion

In this large-scale multicenter study, the clinical and histological features of superficial CRC treated with ESD was reviewed and long-term follow-up outcomes were analyzed. It was observed that the 5-year RFS rate of superficial CRC curatively resected using ESD was 98.8% (338/342), and this result is a favorable outcome. Based on the results, although there was no difference in clinical characteristics between the two groups according to the depth of cancer, it was observed that histological features related to recurrence were significantly higher in SM1 cancer. Nevertheless, the RFS rates were satisfactorily high in both groups (99.3% vs. 97.1%), and no statistically significant difference was found between two groups (*p* = 0.248) (Fig. 3B).

Previous studies have reported favorable long-term outcomes of ESD for CRC [22–26, 30]; however, the prior reported studies had some limitations. The follow-up period was not long enough to confirm the long-term outcomes [23, 25]. The proportion of patients with ECC included in the study was insufficient [23, 25, 26, 30], and patients with high-grade dysplasia were included in the analysis [25]. Therefore, the previously reported studies could not sufficiently represent the long-term outcomes for ECC resected by ESD. In this study, only patients with ECC resected using ESD were evaluated, and a relatively large number of patients were analyzed.

Submucosal lymphatic vessels play an important role in lymphovascular invasion of CRC. Smith et al. reported that submucosal lymphatic vessels were more developed in SM1 than in SM3 colorectal tissue, although there was no difference in vessel size [31]. Consequently, the width of cancer invasion may be associated with greater access to the lymphatic system than the depth, and may subsequently be associated with a higher risk of lymph node metastasis [32]. A submucosal invasion width of more than 11.5 mm or invasion area more than 35 mm^2^ has important implications in the prediction of the presence of lymph node metastasis [33]. In this context, it is necessary to analyze whether the long-term outcomes of SM1 CRC curatively resection with ESD is different from that of intramucosal cancer. This study included many patients with SM1 cancers, enabling statistical analysis of the differences in the characteristics between SM1 and intramucosal cancers. In this study, lymphatic invasion was significantly higher in patients with SM1 cancer. Additionally, lymphatic invasion and other pathological findings related to recurrence were significantly higher. Nevertheless, a low RFS rate was observed, and there was no statistically significant difference in the RFS rate. Based on this study results, SM1 invasion in curatively resected ECC did not seem to have a noticeable effect on the prognosis.

The en bloc resection rate of ESD for superficial CRCs were examined. In this study, the en bloc resection rates for CRC were high [25, 30, 34]. One study showed a nearly 100% en bloc resection rate [20]. In this study, the en bloc resection rate was 85.3%, this was rather low as compared with the results of other studies. This difference was due to the deviation in the enrolled study subjects. Some studies with high en bloc resection rates included adenoma [23] and high-grade dysplasia cases [20]. For large-sized cancer, a part of the submucosal dissection lesion was removed in the middle of the procedure to secure the field of view, and the rest was then dissected. The number of large lesions also affects en bloc resection rate. The 85% is considered to be a sufficiently high level of en bloc rate in ECC with submucosal cancer [26].

Except for the case where metachronous cancer was confirmed, recurrence was confirmed in one case of piecemeal resection, where the resection margin could be evaluated, and in two SM1 cancers. The known significant factors associated with recurrence are granular type laterally spreading tumors, tumor size 40 mm or more, no pre-treatment magnification, more than10 years of experience in conventional endoscopic resection, and piecemeal resection only in ESD [35]. Although this study was a large-scale study, the treatment outcomes of superficial CRC through ESD was excellent, though there were few recurrence cases, and the factors affecting the recurrence could not be identified. If the tumor size is > 40 mm or if piecemeal resection is performed, active surveillance should be considered, even if the ECC is curatively resected. To identify factors associated with poor outcomes in SM1 cancer, additional large-scale studies are required.

The present study had several limitations. First, this was a retrospective study, and it was inherently limited by the retrospective nature of the clinical data collected during the follow-up. Second, although the median follow-up period was relatively short (53.8 months), the follow-up period of most patients was more than 5 years, which was suitable for evaluating long-term prognosis. Additionally, all enrolled patients underwent appropriate follow-up colonoscopy and computed tomography for the evaluation of recurrence for more than 1 year. Third, although the total number of patients was large enough, the small number of recurrent cases may have made the identification of reliable risk factors difficult. Since this study was conducted on patients with early-stage cancer, it is inevitable that there were fewer recurrence cases. Despite these limitations, this study was considered to be sufficient to demonstrate the long-term prognosis of patients with superficial ECC treated with ESD.

In conclusion, patients with curatively resected ECC treated with ESD showed favorable long-term outcomes. SM1 cancer curatively resected by ESD did not show statistically significant differences in RFS rates compared to intramucosal cancer. Based on these results, we suggest that ESD could be considered the first treatment modality for superficial CRC. Since recurrence can be observed even in superficial CRCs completely removed by ESD, further large prospective studies are needed to predict the risk factors associated with recurrence.

## Data Availability

The datasets used and analyzed during the current study are not publicly available due to keeping privacy of patients but are available from the corresponding author on reasonable request.
